# Mannitol Polymorphs as Carrier in DPIs Formulations: Isolation Characterization and Performance

**DOI:** 10.3390/pharmaceutics13081113

**Published:** 2021-07-21

**Authors:** Ayça Altay Benetti, Annalisa Bianchera, Francesca Buttini, Laura Bertocchi, Ruggero Bettini

**Affiliations:** 1 Department of Food and Drug Sciences, University of Parma, Parco Area delle Scienze, Building 8, 43124 Parma, Italy; ayca.altaybenetti@studenti.unipr.it (A.A.B.); annalisa.bianchera@unipr.it (A.B.); francesca.buttini@unipr.it (F.B.); laura.bertocchi@unipr.it (L.B.); 2 Interdepartmental Centre Biopharmanet-Tec, University of Parma, Parco Area delle Scienze, Building 33, 43124 Parma, Italy

**Keywords:** mannitol polymorphs, DPI carrier, aerosolization, salbutamol sulphate, budesonide, inhaler resistance, in vitro toxicity

## Abstract

The search for best performing carriers for dry powder inhalers is getting a great deal of interest to overcome the limitations posed by lactose. The aerosolization of adhesive mixtures between a carrier and a micronized drug is strongly influenced by the carrier solid-state properties. This work aimed at crystallizing kinetically stable D-mannitol polymorphs and at investigating their aerosolization performance when used in adhesive mixtures with two model drugs (salbutamol sulphate, SS, and budesonide, BUD) using a median and median/high resistance inhaler. A further goal was to assess in vitro the cytocompatibility of the produced polymer-doped mannitol polymorphs toward two lung epithelial cell lines. Kinetically stable (up to 12 months under accelerate conditions) α, and δ mannitol forms were crystallized in the presence of 2% *w*/*w* PVA and 1% *w*/*w* PVP respectively. These solid phases were compared with the β form and lactose as references. The solid-state properties of crystallized mannitol significantly affected aerosolization behavior, with the δ form affording the worst fine particle fraction with both the hydrophilic (9.3 and 6.5%) and the lipophilic (19.6 and 32%) model drugs, while α and β forms behaved in the same manner (11–13% for SS; 53–58% for BUD) and better than lactose (8 and 13% for SS; 26 and 39% for BUD). Recrystallized mannitol, but also PVA and PVP, proved to be safe excipients toward lung cell lines. We concluded that, also for mannitol, the physicochemical properties stemming from different crystal structures represent a tool for modulating carrier-drug interaction and, in turn, aerosolization performance.

## 1. Introduction

Dry powder inhalers (DPI) represent, in several cases, a valid alternative to liquid forms for inhalation therapy, namely to nebulizers and pressurized metered-dose inhalers [1,2], due to their more favourable physicochemical stability, and low environmental impact. Usability and simplification of administration manoeuvres and procedures represent further features of DPIs [3]. Good inhalation performance from a DPI includes several aspects, such as reproducibility and adequate availability of the active ingredient at the site of action [4]. These aspects are related to the dispersibility of the powders during the inhalation act and the aerodynamic behaviour of the active pharmaceutical ingredient (API) particles, which are mainly related to particle size distribution and particle density. The large majority of drugs administered in the lungs with DPIs are micronized powders at very low dosage. The most popular approach to increase the performance of these DPIs is founded on carrier-based formulations which imply the formation of an adhesive mixture between the micronized API and a coarse carrier. It is common knowledge that mixture stability, from one side, and the DPI performance, from the other side, result from the balance between adhesive and cohesive forces between the API and carrier [1,5]. Lactose is by far the most commonly used carrier in these applications, although it presents some limitations related to specific categories of lactose-intolerant patients [5]. Moreover, emerging novel APIs may require an increased loading capacity of the carrier [6] or carrier compatibility with APIs carrying primary amino groups such as, for instance, peptides and proteins [5,7]. For these reasons, the search for alternative carriers such as mannitol for DPIs formulations is getting a great deal of interest [4,8,9,10]. In this respect, mannitol is a nonreducing, nonhygroscopic compound showing promising aerosolization properties [5,11,12]. Moreover, its safety on lung administration has been established [13,14].

The interactions between the API and the carrier, as well as those between the API particles in adhesive mixtures, occur at the particle surface and are, therefore, strongly related to the carrier surface characteristics, not only in terms of morphology [15,16,17] but also the solid-state properties. Della Bella et al. [18] demonstrated the strong relationship between lactose crystal phase and aerosolization performance both with hydrophilic and lipophilic model drugs. The role of solid-state properties, such as those induced by grinding/micronization [19], on respirability performance has been evidenced by several other authors [5,8,20,21].

Three main D-mannitol polymorphs, α, β and δ forms [22,23,24], have been isolated and characterized, the β form t being the thermodynamically stable form in standard conditions and, therefore, the commercially available one. Other forms with lower kinetic stability, namely the hydrate and amorphous forms, have also been reported [25].

Not surprisingly, and similar to what is already evidenced for lactose, in the case of mannitol it appears that solid-state properties may play a role in its technological features [26,27,28,29] and aerosolization performance [30]. Therefore, the effect of the crystal phase of D-mannitol used as a carrier in binary adhesive mixtures needs to be addressed in a systematic manner and deserves investigation. To this end, the availability of kinetically stable D-mannitol polymorph powders with particle size distribution suitable for DPI application is a mandatory prerequisite. An interesting cospray drying process for the production of the kinetically stable D-mannitol δ form in the presence of PVP was reported by Vanhoorne and coworkers [31].

The aim of the present work was to set up three easy to scale-up methods for the production of kinetically stable D-mannitol polymorphs to be used as carrier in DPIs by addition of small amounts of hydrophilic polymers, and to characterize their performance on the aerosolization of two model drugs with two different devices. A further goal was to perform a preliminary in vitro assessment of the cytocompatibility of the produced polymer-doped mannitol polymorphs toward Calu3 and A549, which represent two epithelial cell line models for lungs. 

## 2. Materials and Methods

### 2.1. Materials

Pearlitol^®^ 200SD and Pearlitol^®^ 160C were obtained from Roquette, Lestrem, France. Micronized salbutamol sulphate (SS) and budesonide (BUD) were supplied from Fagron, Bologna, Italy and by Chiesi Farmaceutici, Parma, Italy respectively. Lacto-Sphere^®^ MM50 (sieved α-lactose monohydrate, d_V50_ = 53.1 μm) was provided by Micro-Sphere SA (Monteggio, Switzerland). The excipients for the recrystallization phase of mannitol were PVP K30 (Kollidon^®^ 30, BASF, Ludwigshaufen, Germany), PVA 22K (Fluka, Buchs, Switzerland) and CaCl_2_ (Merck, Darmstad, Germany). The analytical method was conducted using acetonitrile (VWR, Milan, Italy) and methanol (VWR, Milan, Italy). The mobile phase and buffer solutions were prepared using K_2_HPO_4_ and KH_2_PO_4_ (both from ACEF, Fiorenzuola, Italy). A 0.45 µm PTFE filter membrane was used (ALBET^®^, Madrid, Spain).

#### 2.1.1. Crystallization Techniques of D-Mannitol

Recrystallization of mannitol was carried out to isolate kinetically stable mannitol polymorphic forms. 

#### α Form Recrystallization

1 g of Pearlitol^®^ 200SD and different quantities of PVA (0, 1 or 2% *w*/*w* of mannitol), were weighed then transferred into an Erlenmeyer flask and brought into solution in 125 mL of methanol on a heated stirring plate at a temperature of 60 °C for about 2 h. The solution was then filtered with a PTFE filter (0.45 μm) using a vacuum pump, and the filtered solution was placed into an ice bath for 30 min under stirring. The obtained mannitol precipitate was finally filtered with a PTFE filter (0.45 μm) and dried in an oven at 30 °C for 12 h [23].

#### Hydrate Form Recrystallization

The hydrate and β forms were prepared by freeze-drying a water solution of Pearlitol^®^ 200SD using a laboratory freeze-drier (Alpha 2–4 LSC Plus, Martin Christ Gefriertrocknungsanlagen GmbH, Osterode am Harz Germany).

The following conditions produced the hydrate form: (i) a D-mannitol solution 4% *w*/*v* in purified water was equilibration at −20 °C for 3 h; (ii) the solution was frozen at −45 °C for 20 min; (iii) primary drying at 0.1 mbar, −15 °C for 2 h, followed by a step at 0 °C for 1 h, then 10 °C for 2 h; (iv) secondary drying at 25 °C for 4 h at 0.1 mbar.

Since this form proved to be not stable enough, as it rapidly dehydrated at ambient temperature forming the β polymorph, an attempt was made to increase its stability by adding a small amount of CaCl_2_ (1% *w*/*w* of mannitol) to the water solution and submitting it to the above-described process of freeze-drying.

#### β Form Recrystallization

The β form of mannitol, which is the thermodynamically stable form, was prepared using two different methods. 

The first was similar to the method used to obtain the hydrated form from freeze drying, but with a longer time (10 h instead of 4 h) in the secondary drying phase (25 °C-0.100 mbar).

The second method involved the use of acetone as an antisolvent. Mannitol Pearlitol^®^160 C (4.5 g) was weighed and dispersed in an Erlenmeyer flask containing 25 mL of ultrapure water to obtain a concentration of 18% *w*/*v*. The solution was magnetically stirred for 2 h until complete mannitol dissolution, and then filtered under vacuum. Acetone (25 mL) was then added to the water solution of mannitol under magnetic stirring for 4 h. The precipitate was filtered using PTFE (0.45 μm) and placed in an oven at 30 °C for 12 h until complete drying [23].

#### δ Form Recrystallization

The δ form was obtained by crystallization in the presence of PVP, thus modifying the method proposed by Cares-Pacheco et al. and Vanhoorne et al. [23,31]. 

Mannitol Pearlitol^®^160 C (4.5 g) was weighed and dispersed in the presence of PVP K30 (from 0.5 to 3% *w*/*w* of mannitol) in 25 mL of ultrapure water in an Erlenmeyer flask. The solution was magnetically stirred for 2 h until complete mannitol dissolution and then filtered (PTFE 0.45 μm) under vacuum. Acetone (25 mL) was then added to the water solution of mannitol under magnetic stirring for 4 min. The obtained mannitol precipitate was finally filtered with PTFE (0.45 μm) and dried in an oven at 30 °C for 12 h [23].

All crystallized mannitol samples were stored in a desiccator with silica gel until further use.

#### 2.1.2. Solid State Characterization

The solid state and physical characteristics of the mannitol phases were assessed by X-Ray diffraction on powders (XRPD), differential scanning calorimetry (DSC), dynamic vapor sorption (DVS), laser light diffraction and scanning electron microscopy (SEM). In order to identify unambiguously the crystalline phase of the recrystallized mannitol, the Cambridge Crystallographic Database was used to obtain the X-ray Powder Diffraction beamline of pure mannitol crystals [22,32]. The diffraction pattern of each pure crystalline phase was obtained by downloading the data file related to the relevant crystal phase in “.cif” format from the Cambridge Crystallographic Database Center (CCDC). This file was processed using Mercury 4.0.0 software (Cambridge Crystallographic Data Centre, Cambridge, UK), to compute the powder pattern of the forms of mannitol. Subsequently, data were converted into Microsoft Excel format and compared to those obtained experimentally.

##### X-ray Diffraction on Powders

The analysis was performed by a MiniFlex diffractometer (Rigaku, Tokyo, Japan) using Cu Kα radiation (λ = 1.5418 Å) generated with 30 kV. The diffractometer accuracy was previously checked using a pure silicon sample (28.4 2*θ* diffraction angle).

Samples were put into the aluminium sample holder until it was completely covered by powder and then compacted with a glass slide to obtain an even surface. The goniometer was set at a scanning rate of 1.5° min^−1^ (step size = 0.05°) over the 2*θ* range 2–35°. Three replicates for each measurement were carried out.

##### Particle Size Distribution

The particle size distribution of the mannitol powders was measured by laser light diffraction (Spraytec, Malvern, UK). Powders were dispersed in cyclohexane at a concentration of 1% *w*/*v* in the presence of 0.1% *w*/*v* of Span^®^ 85 and sonicated for 3 min (8510, Branson Ultrasonics Corporation, Danbury, CT, USA). Particle size distributions for three dispersions of each powder were performed with an obscuration level of at least 10%. Data were expressed in terms of equivalent volume diameter (Dv) for the 10th/50th/90th percentiles of Dv(10), Dv(50), and Dv(90) respectively. The Span value calculated according to Equation (1), was used as a measure of the distribution width:

(1) $$Span=\frac{Dv\left(90\right)-Dv\left(10\right)}{Dv\left(50\right)}$$

##### Differential Scanning Calorimetry

A DSC 821e (Mettler Toledo, Switzerland) driven by STARe software (Mettler Toledo) was employed to investigate the thermal behaviour of mannitol forms in the temperature range between 25 °C and 200 °C at a heating rate of 10 °C/min. For the mannitol δ form, the thermal behaviour was also investigated in more detail in the temperature range between 120 °C and 200 °C with a heating rate of 5 °C/min.

The instrument was previously calibrated with Indium (onset of melting T_m_ = 156.48 °C, enthalpy of melting ΔH_m_ = 28.60 J g^−1^).

Samples of about 5–8 mg were placed in a 40 µL aluminium pan with a pierced cover and heated under a flux of dry nitrogen (100 mL/min).

##### Thermogravimetric Calorimetry

Thermogravimetric analyses performed with the TGA/DSC1 instrument (Mettler Toledo, Greifensee, Switzerland) were used to evaluate the water content of the powders based on their weight loss due to heating. Samples of about 5–8 mg were weighed in 40 µL aluminium pan and heated in the temperature range between 25 °C and 100 °C at a heating rate of 10 °C/min under nitrogen flow at 80 mL/min.

##### Dynamic Vapor Sorption

The water absorption and stability of the mannitol forms were assessed with an Aquadyne DVS-2 (Quantachrome Instruments, Baynton Beach, FL, USA) using a gravimetric approach. The instrument was calibrated in 0–90% relative humidity (RH) range at 25 °C with a certified standard of microcrystalline cellulose (microcrystalline cellulose for water sorption isotherm measurements, CRM n. 302, individual identification n. 0441, E.U. Bureau of reference). The balances of the instrument were calibrated at 25 °C, 50% RH using a 200 mg standard weight prior to the measurement of each specimen. The mannitol samples (about 50 mg) were analysed at 25 °C with 5% step of relative humidity (RH) increase from 5% to 95% followed by a decrease in the reverse order. The analysis was initiated by a drying step at 60 °C and 0% RH for 30 min to eliminate any residual moisture from the surface of the sample. The transition from one step to the next occurred when the rate of weight variation was lower than 0.001% min^−1^ and, in any case, not earlier than 20 min from the beginning of the step.

##### Scanning Electron Microscopy

The morphology of the different mannitol polymorphs was visually investigated via scanning electron microscopy using a FESEM SUPRA™ 40 (Carl Zeiss, Jena, Germany). Each powder sample was deposited on adhesive black carbon tabs premounted on aluminium stubs to allow the dispersion of the charge and coated with a gold film of about 60 nm. The particles in excess were gently removed with a nitrogen flow. The samples were analysed under high vacuum conditions (1.33 × 10^−2^ Pa for 30 min) and the images collected at different magnifications using an accelerating voltage of 1 kV.

#### 2.1.3. Preparation of Adhesive Mixtures

All blends were prepared with 1% drug content of the final weight. Two model drugs were selected, namely salbutamol sulphate (SS) and budesonide (BUD). To assess the better and faster method of preparation, the “sandwich” (or layering, which consists of placing a fraction of the excipients in the blender, then layering the active ingredient over the surface of the excipient, and finally putting the remaining part of the excipient on the top of the powder bed) and the geometric dilution methods were compared. The same mixture was prepared by the two methods using a Turbula^®^ blender, and five samples of each mixture were collected every five minutes to be analysed via HPLC. The mixture was considered homogeneous when the coefficient of variation (calculated as the percentage of the ratio between standard deviation and mean value of the five measurements) was lower than 5%. The geometric dilutions method ensured better yield and shorter preparation time. Therefore, 2 g of each mixture (model drug and different mannitol polymorphs) were prepared using this method by mixing for one hour at 40 rpm.

#### 2.1.4. In Vitro Aerodynamic Property Assessment

The aerodynamic assessment of the blends was performed using an Andersen Cascade Impactor (ACI; Copley Scientific, Nottingham, UK) employing two devices with different resistance RS01^®^ (RPC Plastiape Spa, Osnago, Italy) and the prototype NESAT^®^ (Bormioli Pharma Srl, Parma, Italy). The effective cut-off diameters (ECD) of each stage were then recalculated according to Stokes’s law using the ECDs referring to a flow of 28.3 L/min [20]:

(2) $$D_{Q}=D_{28.3}\sqrt{\frac{28.3}{Q}}$$

where *D* refers to the cut-off diameter at the flow *Q* used during in vitro test. 

The preseparator, having a 10 µm of cut off diameter, was equipped with a liquid trap (10 mL of ultrapure water) in order to capture noninhalable large particles. The micro-orifice collector (MOC) was equipped with a type A/E glass filter (Whatman, Little Chalfont, UK). To avoid particle bounce, ACI plates were coated with 1% (*w*/*v*) glycerol in methanol for SS and 2% (*w*/*v*) Tween 20 in ethanol for BUD, allowing solvent evaporation before ACI assembling. The device was connected by a rubber mouthpiece adapter to the ACI, and an air stream generated by a VP 1000 vacuum pump (Erweka, Langen, Germany) was set to have a pressure drop behind the impactor of 4 kPa and 4 L of the air volume. Thus, 4 s at a flowrate of 60 L/min or 5.1 s at a flowrate of 48 L/min were used for RS01^®^ and NESAT^®^, respectively. The device resistance, *R*, could be calculated as:

(3) $$R=\frac{\sqrt{Pressure\;drop\;\left(\mathrm{kPa}\right)}}{Flowrate\;\left(L\;\min^{-1}\right)}$$

This resulted in a medium/high value for NESAT and a medium value for RS01. 

Size 3 capsules (Quali-V^®^ Qualicaps Europe, Madrid, Spain) were manually filled with 20.0 ± 0.2 mg of powder. After the aerosolization process, the capsule was removed from the device and the deposition test repeated until six capsules had been discharged. At the end of the test, the drug deposited at the various levels (capsule and device, rubber and throat, preseparator stage 0, 1, 2, 3, 4, 5, 6, 7 and filter) was collected with a mixture of acetonitrile:water (6:4 *v*/*v*) for BUD and with distilled water for SS. The residual part of drug in the capsule device, rubber adaptor, throat and pre-separator were collected individually in 50 mL flasks, whereas the drugs deposited on the plates and filter were washed in crystallizers containing 10 mL of solvent. The drug solutions obtained by washing the capsule, device and filter were also sonicated for 5 min and filtered with RC filters to ensure homogeneity. The concentration of SS and BUD in each sample was determined by HPLC analysis. Each formulation was tested three times.

The obtained data were processed with Microsoft Office Excel 16.16.21 (Microsoft Corp., Redmond, WA, USA) and KaleidaGraph (version 4.5.4 Sinergy Software, Washington, DC, USA) software to obtain aerodynamic parameters, i.e., the emitted dose (ED) which is the amount of drug discharged from the device after inhalation; the emitted fraction (EF%), calculated as the ratio between the emitted dose and the loaded dose in the capsule, the fine particle dose (FPD) that corresponded to the amount of drug recovered in the stages of impactor, the fine particle fraction (FPF%) calculated as the ratio between fine particle dose and emitted dose and the mass median aerodynamic diameter (MMAD), which is the diameter that separates the aerosolized particles in two populations with equal weight. MMAD was determined by plotting the cumulative percentage of mass less than the cut-off diameter for each stage on a probability scale versus the relevant aerodynamic diameter of the stage on a logarithmic scale. MMAD corresponds to the slope of the line obtained by linear regression of the experimental points.

#### 2.1.5. High Performance Liquid Chromatography (HPLC)

The samples collected from aerodynamic studies were loaded into vials for HPLC analysis conducted using an Agilent 1200 LC Series (Agilent Technologies, Santa Clara, CA, USA), driven by ChemStation software A.04.02 using a UV detector set at wavelengths of 220 and 254 nm for salbutamol sulphate and budesonide, respectively. The applied analytical methods differed between budesonide and salbutamol. For salbutamol sulphate, the mobile phase was prepared from 6 g of KH_2_PO_4_ in 800 mL of ultrapure water. After neutralizing to pH 7 with 10 M NaOH, the solution was brought to a volume of 1 L and filtered with a 0.45 μm CA-filter (Sartorius Stedim Biotech GmbH, Göttingen, Germany). The obtained phosphate buffer was mixed with methanol (4:6 *v*/*v*) to obtain the final mobile phase that was pumped at 0.6 mL/min, through a Supelcosil^TM^ LC−18 column (25 cm × 4.6 mm, 5 μm) kept at 30 °C; 50 μL of sample solution were injected and the retention time was 5.6 min. The analytical method was assessed in terms of linearity of response (AUC vs. concentration) in the concentration range 4–40 μg/mL. Limit of Quantification, LOQ = 1.04 μg/mL; Limit of Detection, LOD = 0.31 μg/mL were evaluated as:

LOD = 3.3 σ/*slp* (4)

LOQ = 10 σ/*slp* (5)

Here σ and the *slp* are the standard deviation and the slope, respectively, of the regression line of the absorbance vs. concentration experimental points. For budesonide, the mobile phase was a mixture of acetonitrile: water (6:4 *v*/*v*). The flow was set at 0.75 mL/min through an Atlantis^®^ dC18 column (150 mm × 3.9 mm), 50 μL of sample solution was injected and the retention time was 3.3 min. In this case the analytical method was assessed in terms of linearity of response in the concentration range 4–40 μg/mL (LOQ = 0.32 μg/mL; LOD = 0.097 μg/mL).

#### 2.1.6. Preliminary Cell Toxicity

Cell viability was measured in terms of mitochondrial activity by using the 3-(4,5-dimethylthiazol-2-yl)-2,5-dyphenyltetrazolium bromide (MTT) assay on a human lung cancer cell line, Calu3 (ATCC^®^ HTB-55^®^) [32], and epithelial carcinoma cell line, A549 (ATCC^®^ CCL-185^TM^). Cells were seeded into 96-well plates (VWR Tissue culture plates, VWR International, Italy) at a density of 3 × 10^4^ cells/well for Calu3, or 1 × 10^4^ cells/well, for A549, in 200 μL of culture medium composed of MEM (minimum essential medium, Gibco^®^, Thermo Fisher Scientific, Waltham, MA, USA) with the addition of 10% fetal bovine serum (FBS, Heat inactivated, Aurogene s.r.l., Rome, Italy) 1% penicillin/streptomycin solution (100× Aurogene s.r.l., Rome, Italy) and 1% of nonessential amino acid solution (MEM NEAA, Gibco^®^, Thermo Fisher Scientific, Waltham, MA, USA). Cells were left to settle overnight before performing the viability assay.

The powder samples were dissolved in Hanks buffered salt solution (HBSS, Gibco, Thermo Fisher scientific, Waltham, MA, USA) +30 mM HEPES (>99.5% H3375, Sigma-Aldrich, St Louis, MO, USA). Before the test, the growth medium was removed and 150 μL of each solution to be tested was added to each well and left for 24 h at 37 °C at 5% CO_2_. After incubation, solutions were gently removed and 150 μL of 1 mg/mL solution of thyazol blue tetrazolium bromide (M2128, Sigma-Aldrich, St Louis, MO, USA) in HBSS+ 30 mM HEPES was added and left for 2 h at 37 °C at 5% CO_2_. After removing the solution, precipitated formazan crystals were dissolved in 100 μL DMSO for each well, with shaking, for 10 min in the dark. Then, the absorbance of the samples was read at 570 nm by means of a plate reader Spark^®^ (Tecan, Männedorf, Switzerland). The concentrations of mannitol, PVP or PVA to be tested were chosen on the basis of a rough estimate of the amount of each component, in terms of respirable fraction, that could reach the lungs following an inspiration from a capsule loaded with about 20 mg of excipient, which was set at 2 mg. This amount is supposed to be dissolved in the available volume of lung lining fluid estimated between 10 and 30 mL [33]. On these bases and considering the worst-case scenario (more concentrated solution), 0.2 mg/mL mannitol was considered as the reference concentration to be evaluated, and a viability assay was executed over eight concentrations following a serial dilution of two covering and exceeding this value. As a consequence, PVP and PVA, when taken alone, were tested at concentrations reflecting their relative ratios with mannitol during crystallization, namely 1% *w*/*w* and 2% *w*/*w,* respectively. A final reference concentration of 2 μg/mL and 4 μg/mL. HBSS + 30 mM HEPES was chosen as a solvent for powder as well as negative control for the test. The viability of cells was expressed as a percentage with respect to untreated negative control as mean value + standard deviation (*n* = 6).

The statistical analysis was performed with Microsoft Office Excel 16.16.21 software employing a two-tailed unpaired *t*-test with significance level fixed at *p*-value = 0.05. Standard deviation was used to indicate experimental variability.

## 3. Results

### 3.1. Solid-State and Physical Characteristics Assessment of Mannitol Crystallized Forms

The purpose of this work was to investigate whether mannitol powders in different crystal phases could affect the aerosolization behaviour of two model active pharmaceutical ingredients when the mannitol particles were used as carriers in an adhesive mixture. Previous work with lactose as a carrier [18] clearly evidenced the influence of the lactose crystal phase on aerosolization performance both with hydrophilic and lipophilic model API. To accomplish this aim, the availability of pure solid phases of mannitol represents a fundamental prerequisite.

Starting from the work of Cares-Pacheco et al. [23] (for the α form) and Vanhoorne et al. [29,33] (for the δ form), modified crystallization procedures were developed in order to isolate four mannitol solid phases suitable for the production of binary mixtures with the selected micronized API in terms of kinetic stability and physical characteristics such as shape and particle size distribution.

**Figure 1:**
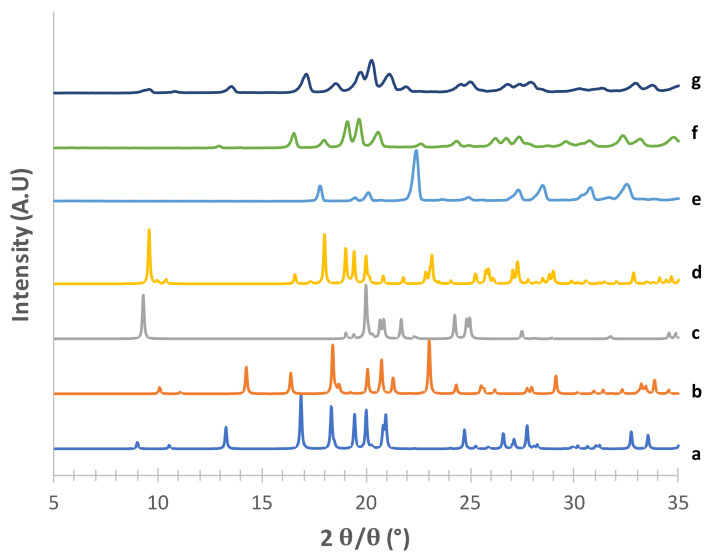
PXRD patterns of the supposed mannitol α form (g) prepared in a saturated methanol solution using 2% of PVA (f) 1% of PVA, (e) without PVA. Patterns of all polymorphs are added as references (a) α form CCDC 224658, (b) β form CCDC 224659, (c) δ form CCDC 224660, (d) hemihydrate form CCDC 251528.

**Figure 2:**
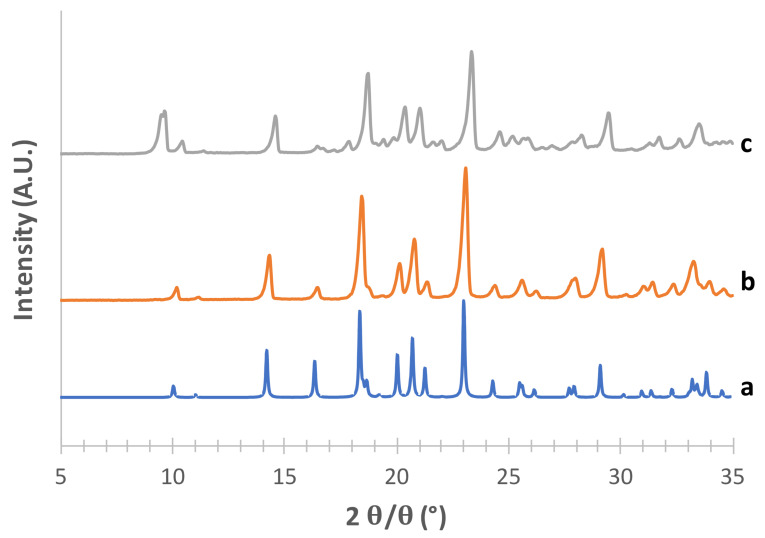
PXRD patterns of mannitol β form (c) obtained using acetone as an antisolvent. (b) By freeze drying technique; (a) CCDC 224659 as a reference.

**Figure 3:**
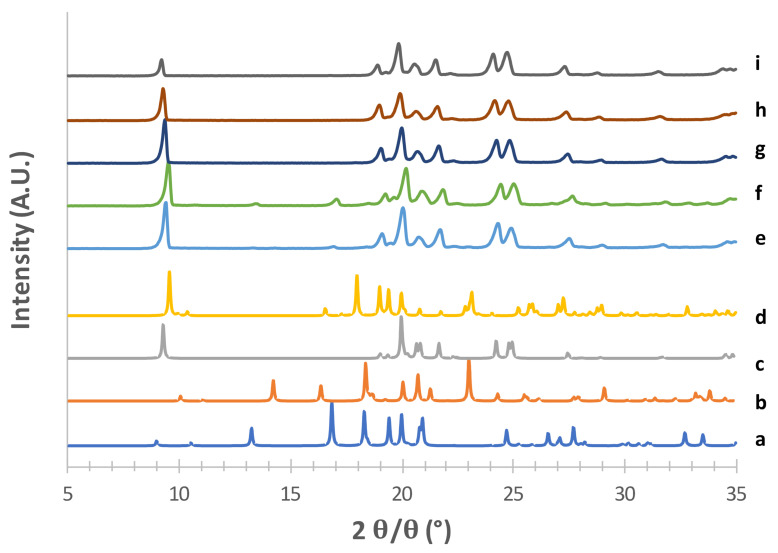
PXRD patterns of mannitol δ form (i) obtained using acetone as an antisolvent with 3% PVP (h), 2% PVP (g), 1% PVP (f), 0.5% PVP (e) without PVP. Patterns of all polymorphs are added as references (a) α form CCDC 224658; (b) β form CCDC 224659; (c) δ form CCDC 224660; (d) hemihydrate form CCDC 251528.

**Figure 4:**
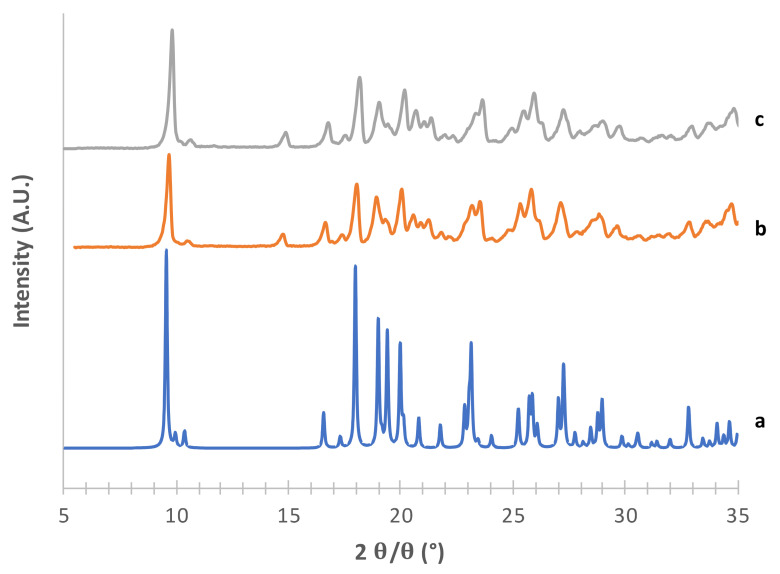
PXRD patterns of mannitol hemihydrate forms (c) obtained by freeze drying using 1% of CaCl_2_ (b) without any additional component in mannitol water solution (a) CCDC 251528 [25] as a reference.

The obtained mannitol solid phases were, first of all, unequivocally identified by comparing their powder X-ray diffraction patterns with those relevant to β, δ, α and hydrate forms obtained from the CCDC [22,25]. Figure 1, Figure 2, Figure 3 and Figure 4 show the PXRD patterns of the crystallized mannitol β, δ, α and hydrate forms in comparison with the relevant theoretical form from CCDC. 

Considering the supposed α form (Figure 1) in the absence of PVA (e) [23], the diffraction pattern substantially differed from that obtained from CCDC (deposition number 224,658, [22]). The positions of the observed peaks, and in particular the presence of a peak at around 2*θ* = 22.6°, suggest that in this condition we obtained a mixture of α and β forms. The addition of 2% PVA (g) resulted in good matching with the reconstructed pattern, as testified in particular by the presence of the diagnostic peaks at 2*θ* = 8.91°, 2*θ* = 10.45°, 2*θ* = 13.17° and the most intense peak at 2*θ* = 16.77°. At 1% PVA (f) the presence of the latter peak at low intensity suggests the formation of a mixture of phases rather than the pure α form, as also indicated by the absence of the peaks at 2*θ* = 8.91° and 2*θ* = 10.45° (see Supplementary Table S1). Thus, 2% PVA appears to be the lower polymer concentration suitable for obtaining the pure α form. Although the use PVA in formulation for inhalation has already been proposed [34] it is worth underlying that PVA has not yet been approved for such applications. Therefore, a low concentration of PVA in the carrier would be beneficial for reducing the polymer burden that may reach the respiratory tree, although it should be considered that most of the carrier deposits in the oropharyngeal region and is eventually swallowed.

As for the supposed β form (Figure 2), the superimposition with the pattern reconstructed from CCDC data (deposition number 224,659, [35]) was quite good for the powders obtained with both crystallization methods, in particular for the characteristic peaks at 2*θ* = 10.11°, 2*θ* = 14.27°, the most intense peak at 2*θ* = 23.07° and the peak at 2*θ* = 20.09° (see Supplementary Table S2).

The PXRD pattern obtained from CCDC for the supposed δ form (deposition number 224,660, [35]) is shown in Figure 3 (c) along with those obtained from the powders prepared by modifying the method proposed by Vanhoorne et al. [26,31] who added 5% of PVP to obtain the stable mannitol δ form. Similar to the approach adopted for the α form, the rationale here was to figure out the lowest amount of polymer necessary to obtain the crystallographic pure (and possibly kinetically stable) δ form in order to minimize the polymer potentially reaching the lower airways. Thus, five different PVP concentrations (from 0 to 3% *w*/*w* of mannitol) were investigated (Figure 3). Without PVP (e), as well as with 0.5% *w*/*w* polymer (f), the highly intense peak at 2*θ* = 9.35°, which is characteristic of the δ form, was observed. However, in the trace from CCDC (c) no peaks were present in 2*θ* region between 9.5–18.5°, whereas 0 and 0.5% PVP-containing powders showed small peaks at 2*θ* = 13.43° and 2*θ* = 17.05°, likely ascribable to the presence of traces of the α form (see Supplementary Table S3). These two peaks disappeared as the PVP concentration increased, 1% *w*/*w* PVP (g) being the minimum polymer concentration to obtain the crystallographically pure δ form. This powder did not differ from those containing 2% (h) and 3% PVP (i).

The effect of PVP doping on the mannitol solid-state was also investigated by DSC (Figure S1 in Supplementary material), which showed the appearance of the melting endotherm of the δ mannitol form (onset melting temperature of 155.4 °C at a peak of 157.2 °C) starting from 1% PVP.

It is worth underscoring that the amount of polymer added in the case of PVA and PVP was below the sensitivity of the diffractometer. Therefore, it was not possible to speculate about the solid-state characteristics of the polymer in the carrier particles. Nevertheless, the unidentified small peaks observed in Figure 1 and Figure 3 could be reasonably attributed to the presence of either PVA or PVP in crystalline form, respectively.

The hydrate form of mannitol was obtained by freeze-drying with final drying at 25 °C for 4 h at 0.1 mbar [36]. A hemihydrate solid phase was isolated (Figure 4), although not in a pure form (see extra reflection at around 15° 2*θ* and Supplementary Table S4)*).* However this form was not stable enough, as it easily dehydrated at room temperature, converting into the anhydrous form [25]. For this reason, its crystalline form was characterized, but it was not used for the preparation of adhesive mixtures with the drug used in the second phase of the work.

**Figure 5:**
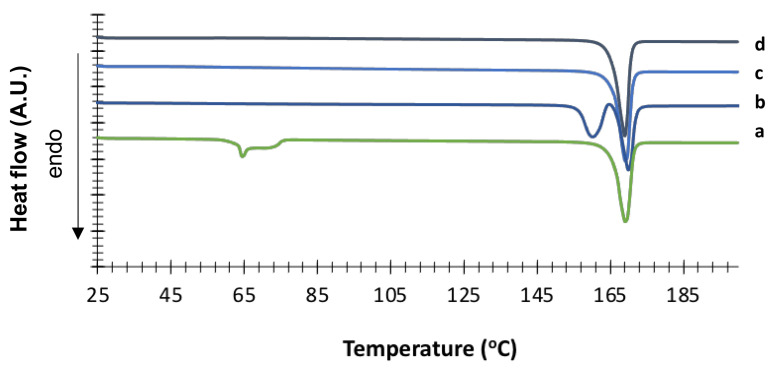
DSC traces of crystallized mannitol forms: (a) hydrate; (b) δ form (1% PVP); (c) β form; (d) α form (2% PVA).

The hydrate nature of the crystal was confirmed by DSC (Figure 5), which showed a fairly broad endothermic event (onset 62.9 °C ± 0.1 °C, peak 64.0 °C ± 0.1 °C) ascribable to the pseudopolymorphic transition (release of water molecule from the hydrate crystal) followed by final melting with onset at 164.5 °C ± 0.2 °C. TGA analysis indicated a weight loss of 5.2% ± 0.5% in the temperature range 25–80 °C, thus confirming the loss of half a molecule of water per molecule of mannitol (theoretical value 4.7% *w*/*w*) (Figure S2 in Supplementary Materials).

For comparison purpose, Figure 5 also shows the DSC traces of the other crystallized mannitol forms. δ mannitol showed an onset melting temperature of 155.4 °C ± 0.4 °C with a peak at 157.1 °C ± 0.2 °C. This endothermic event was followed by a small exotherm ascribable to recrystallization, and a final melting at 165.2 °C ± 0.5 °C (onset). As for α mannitol, the onset melting temperature was t 164.4 °C ± 0.1 °C with the peak at 165.6 °C ± 0.1 °C. The β form showed an onset temperature of fusion at 165.2 °C ± 0.5 °C with the peak at 166.0 °C ± 0.5 °C. These data are in good agreement with those reported by Cares-Pacheco and coworkers [23].

### 3.2. Stability of Crystallized α and δ Mannitol

**Figure 6:**
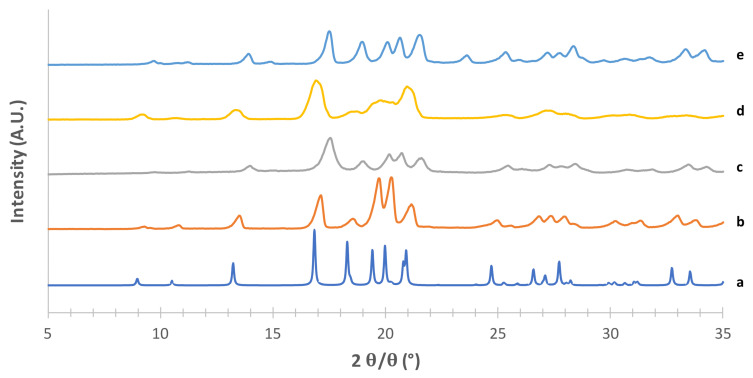
PXRD patterns of mannitol α form recorded at (b) time zero; (c) 1 month; (d) 12 months (e) 24 months storage at 40 °C and 75% R.H. (a) CCDC 224658 as reference.

**Figure 7:**
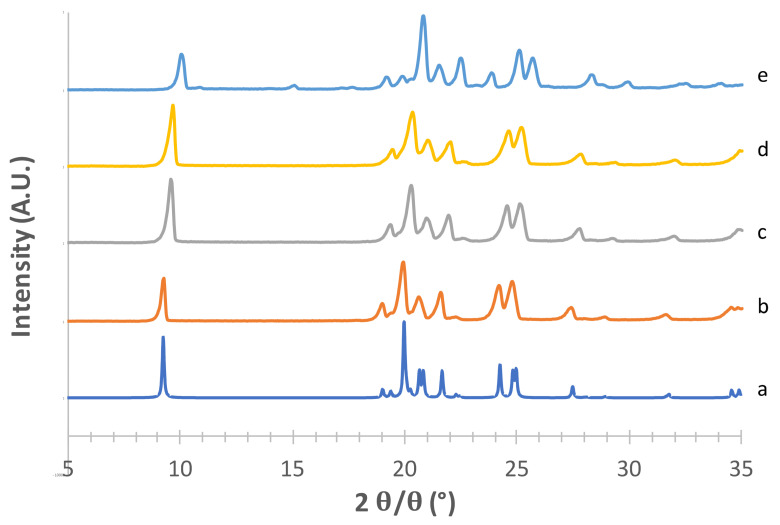
PXRD patterns of mannitol δ form recorded at (b) time zero; (c) 1 month; (d) 12 months; (e) 24 months storage at 40 °C and 75% R.H. (a) CCDC 224660 as reference.

XRPD was used to study the stability of α and δ forms over time (Figure 6 and Figure 7, respectively). The crystallized powders were stored at a temperature of 40° C and 75% RH for up to 24 months in glass vials stoppered with elastomeric caps and clamped. 

XRPD analyses were performed to evidence possible solid-phase changes.

As for the α form, although the peaks appeared slightly translated from one time sample to another (likely due to preferential orientation phenomena stemming from slight particle size distribution differences among specimens), the patterns were substantially similar, as no new peaks appeared within 12 months. However, 24 months storage resulted in small peaks at 2*θ* = 15° and 2*θ* = 23.6°, likely ascribable to the presence of traces of the β form (see Supplementary Table S5).

In the case of the δ form, the patterns recorded after one month or 12 months storage were practically superimposed to that recorded at time zero. After 24 months storage, small peaks were detected at 2*θ* = 10.88°, 2*θ* = 15.08° and 2*θ* = 23.85°, attributable to the presence of traces of the β form (see Supplementary Table S6).

Therefore, it can be stated that both polymorphic forms kept their solid-state characteristics for at least 12 months under accelerated stability conditions and likely for longer time at ambient conditions.

The stability of the δ form crystallized with different concentrations of PVP was also evaluated by DVS (Figure S3 in Supplementary Materials). The PVP-free powder presented an isothermal change in weight of only 1% in the 5–95% RH interval. Powders containing higher concentrations of PVP tended to interact more with water vapor, showing a maximum of 2% of weight increase in the case of the powder containing 3% PVP. This was not unexpected given the hydrophilic nature of the polymer. Interestingly, none of the tested samples gave rise to hysteresis, suggesting reversible interaction with humidity as also indicated by the DSC traces recorded on the samples after DVS experiments (Figure S4 in Supplementary Materials) that were practically superimposable on those recorded before humidity exposure.

### 3.3. Particle Size Distribution and Morphology

The particle size distribution (PSD) of the powders of the recrystallized mannitol polymorphs, determined by laser light scattering, is summarized in Table 1.

The β form showed the narrowest particle size distribution with the majority of the particle having a size lower than 30 µm, while α and δ forms (as well as the hydrate) showed similar PSD with a median volume diameter around 23 µm. In view of a possible use of these powders as carriers in adhesive mixtures, it must be underscored that these figures are low compared to the typical median diameters of lactose carriers, which range between 10 and 150 µm, although specific lactose for inhalation, such as Respitose^®^ ML006 [37] having a PSD very similar to that of the mannitol particles reported here are available on the market.

**Figure 8:**
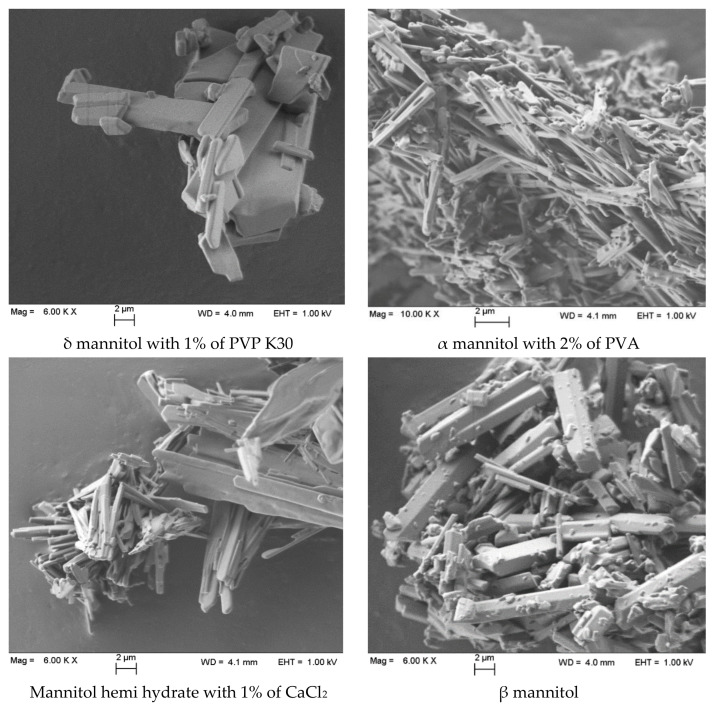
SEM pictures of mannitol forms taken at 6000× (or 10,000× in the case of α form) magnification.

The morphology of the crystallized mannitol particles was assessed by scanning electron microscopy. Example SEM pictures of the different forms are presented in Figure 8. In all cases, prismatic crystals were obtained with the α form showing a higher number of fine particulates aggregated to form threadlike crystals. The β form showed more variable shape distribution with several elongated and needle-shaped crystals.

### 3.4. Adhesive Mixtures

Adhesive mixtures of two model API with different mannitol forms and lactose MM50 as the reference carrier were prepared to study their aerosolization performance with medium (RS01^®^) and medium-high (NESAT^®^) resistance device.

Table 2 reports the actual drug content and the loaded powder doses in the capsule for each mixture.

### 3.5. Salbutamol Sulphate Deposition

Table 3 reports the aerosolization parameters of SS from adhesive mixtures prepared with α, β and δ mannitol polymorphs in comparison with those obtained from lactose MM50, aerosolized with RS01 device.

Different mannitol forms lead to different SS deposition profiles. The mixtures prepared with the β form afforded an almost complete delivery of the loaded dose (97.7%), while with the powders having the α form and lactose, almost 20% of the powder dose was retained in the inhaler device and in the capsule. Nevertheless, the emitted dose was high (>80%) in all cases. In terms of FPF, there was no significant difference between the α and β form-containing mixtures, which afforded values around 13%, while the mixture with the δ form gave rise to a lower deposition of fine particles on the impactor (*p* = 0.01 compared to both formulations with the α and β forms).

Despite the high emitted dose, most of the emitted powder was constituted by a coarse fraction as indicated by the percentage of the loaded dose deposited in the pre-separator: 38.64% ± 12.8% for blends with lactose, 47.41% ± 3.73% for the formulations with the δ form and 47.98% ± 14.23% and 51.81% ± 1.24% for the formulations with the α and β form, respectively.

When aerosolization was performed with the NESAT^®^ device (Table 4), a trend similar to that already observed with RS01 was noticed. In general, the percentage of emitted dose was higher than with RS01, except for the mixture with mannitol β form, although the difference was not statistically significant. A better dispersibility of the lactose formulation was obtained with NESAT^®^ compared to RS01 (FPF 13.5 vs. 8.4).

On the other hand, as for the case of aerosolization with RS01^®^, it was confirmed that among mannitol polymorphs, the δ forms gave rise to adhesive mixtures with SS behaving in different manners and affording lower FPF (*p* = 0.04, *p* = 0.01 vs. β and α form, respectively).

The α and β forms had similar dispersibility regardless of the devices used, while, as already stated, some effect of the device type and resistance (although not dramatic) could be observed for formulations prepared with lactose MM50 and the mannitol δ form (increased dispersibility for lactose and decreased for mannitol δ passing from RS01 to NESAT).

A study from Kaialy and the coworkers [8] indicated that there was a significant difference between recrystallised and commercial (β form) mannitol in terms of aerodynamic performances when salbutamol sulphate was used as a model drug. According to their in vitro aerodynamic test via multi-stage liquid impinger, FPF values of the SS blend with recrystalised mannitol were considerably higher than FPF of the SS blend with commercial mannitol [8]. However, the investigation of the solid-state of their recrystalised mannitol showed that it was a mixture of α, β and δ forms and not a single solid phase. On the other hand, the study of Boshhiha and Urbanetz showed that no differences could be evidenced between commercial lactose and mannitol when SS was used as a model drug [38].

Here we report a significant difference in FPF between lactose and mannitol both in the β and α form despite a nonsignificantly different MMAD.

### 3.6. Budesonide Deposition

The values relevant to the aerosolization performance of budesonide with RS01^®^ and NESAT^®^ devices are reported in Table 5 and Table 6, respectively. With both devices, trends similar to those already presented for SS were observed, with the only significant difference that, in the case of BUD, the values of FPD and FPF were much higher than those recorded for SS, while the MMADs were significantly smaller.

In detail, the BUD deposition profile from the RS01^®^ device were not significantly different between the α and β form, while the mixture BUD with the δ form resulted in a significantly lower deposition of fine particles on the impactor. The deposition profile from the MM50 mixture was significantly lower in terms of the fine particle fraction compared to the blends with the α and β forms (*p* = 0.02, *p* = 0.004, respectively). These results are partly in agreement with those reported in the study of Nokhodchi and coworkers who showed that the highest percentage FPF of budesonide was obtained with the formulation of mannitol compared to other carriers, and attributed this better aerosolization performance to the elongated shape of the carrier rather than to its specific surface chemistry [8,35].

With the NESAT^®^ device, very similar figures were observed except for the performance of the mixture prepared with the mannitol α form that afforded significantly higher FPD and FPF relative to RS01^®^.

These results clearly indicate that the manipulation of the solid-state of mannitol can have an impact on the in vitro deposition of two model drugs in relevant adhesive mixtures. Formulations prepared with α and β polymorphs behaved similarly affording better aerosolization performance with respect to the formulations prepared with δ mannitol polymorphs or the reference lactose MM50. In the case of the β form, the better performance might be ascribed to the small particle size distribution and, in particular, to the greater percentage of micronized particles (see Table 1). In fact, in the case mixtures with lactose, it is common knowledge that the presence of fine particles improves the aerosolization performance [5,18,39]. However, this was not the case for the mixture prepared with mannitol α polymorphs that showed a particle size distribution practically superimposable to that of mannitol form δ. Thus, the differences observed among mannitol polymorphs performance may more likely be ascribed to the different characteristics of the particle solid state rather than to a physical property such as size, whereas this latter cannot be excluded *a priori* when considering the comparison with lactose (Dv50 = 53 µm vs. nearly 23.05 µm for α and δ mannitol). 

A final point worth mentioning is the role of the device resistance on the aerosolization performance. As already highlighted, this did not seem to play a significant role in the case of adhesive mixtures prepared with the various mannitol forms containing either SS or BUD; however, mixtures prepared with lactose MM50 deserve particular attention. Similar mixtures with SS and BUD were also investigated by Della Bella et al. [18] using a Turbospin^®^ device at a flow of 70 Lmin^−1^. Therefore, by combining the data reported in this work with those of Della Bella et al., it is possible to study the correlation between resistance of the device (calculated according to Equation (3)) and FPF for both SS and BUD (Figure S4). In agreement to data reported by Hassoun et al. [37], no correlation seemed to exist for the hydrophilic model drug SS (Figure S4, panel a), while a clear linear correlation (R^2^ = 0.997) was highlighted for the lipophilic drug BUD (Figure S4 panel b).

### 3.7. Preliminary Cell Toxicity Evaluation

As previously stated, although the use of PVA in a specific formulation for inhalation has been proposed [34], no data have been reported about compatibility of this excipient toward lung epithelial cells. In addition, it is worth underlying that that neither PVA nor PVP have yet been approved as excipients for inhalation. Moreover, taking into consideration the particle size distribution of the mannitol powders proposed here as carriers for inhalation (Table 1), it would not be unlikely that some small carrier particles would reach the lung while the biggest ones would deposit in the oropharynx and be swallowed. Thus, it is worth raising a concern about the possible toxic effect of the two polymers at the adopted concentration in the mannitol carrier.

As reported from our experiments, the concentrations of mannitol, PVP or PVA to be tested were based on the estimate of the amount of each component potentially reaching the lungs following an inspiration from a capsule loaded with about 20 mg of excipient, which, on the base of the in vitro aerosolization results, was set at 2 mg. It was assumed that this amount would dissolve in the available volume of the lung lining fluid, which has been reported to range between 10 and 30 mL [33]. A worst-case scenario (more concentrated solution) was assumed; thus, 0.2 mg/mL mannitol was considered as reference concentration to be evaluated in the cell viability assay. PVP and PVA alone were taken as references and tested at concentrations of 2 μg/mL and 4 μg/mL, respectively, reflecting their relative ratios with mannitol in the carrier particle (1% *w*/*w* and 2% *w*/*w*, respectively).

**Figure 9:**
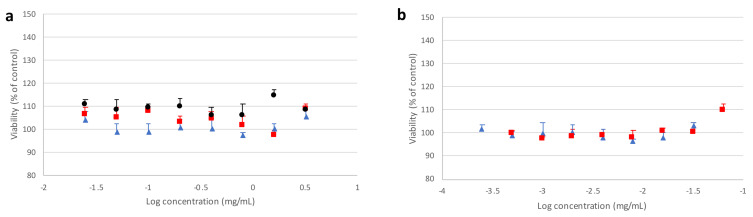
Viability of Calu3 cells after 24 h of contact with (**a**) mannitol polymorphs α (red square), β (black circle), δ (blue triangle), or (**b**) corresponding nominal amounts of doping agents PVA (red square) or PVP (blue triangle). The bars represent standard deviation (*n* = 6).

**Figure 10:**
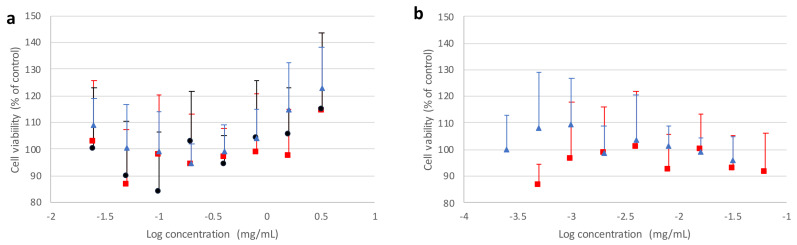
Viability of A549 cells after 24 h of contact with (**a**) mannitol polymorphs α (red square), β (black circle), δ (blue triangle), or (**b**) corresponding nominal amounts of doping agents PVA (red square) or PVP (blue triangle). The bars represent standard deviation (*n* = 6).

Figure 9 and Figure 10 report, respectively, cell viability values obtained from the MTT assay after incubating Calu3 or A549 cell lines in the presence of different forms of mannitol for 24 h, as well as PVP and PVA alone, with respect to the untreated control.

It can be observed that the crystallized mannitol polymorphs had no deleterious effect on cell viability, as no significant difference could be detected between control cells and treated cells at all concentrations considered, up to 8 times above the reference value (0.2 mg/mL), which was estimated as a “worst case scenario” concentration. For A549 cell line (Figure 10), a generally higher variability was observed on viability, which never fell below 85% with respect to control and increased with higher concentrations of mannitol polymorphs, confirming that no cytotoxic effects were ascribable to excipients. These results suggest that not only the recrystallized mannitol, but also PVA and PVP, could be considered as relatively safe excipients for lung administration, thus adding further support to data previously reported in the literature for PVA [40,41] and PVP [42,43,44].

## 4. Conclusions

Mannitol polymorphs can be produced with simple processes which involve doping with small amounts of selected hydrophilic polymers. The anhydrous polymorphic metastable forms α and δ are kinetically stable for at least one year under accelerated conditions, which suggests a longer stability time at ambient temperature and their possible use as carriers for the preparation of binary adhesive mixture for pulmonary delivery of low-dose micronized active ingredients. The pseudopolymorphic form, namely mannitol hemihydrate, on the contrary, tends very rapidly to convert into the β form, which represents the thermodynamically stable form at standard conditions.

Although the particle size distribution of the crystallized mannitol polymorphs was not optimized, the aerosolization performance of the mixtures prepared with the α and β mannitol crystal forms proved to be superior, or comparable, to that of the mixture prepared with lactose, for both the model drug tested and with either a medium or medium/high resistance inhaler.

As for lactose, mannitol had better aerosolization performance with the lipophilic model active ingredient compared to hydrophilic ingredient.

Different from lactose, the aerosolization of the best performing mannitol polymorphs was not affected by the device resistance.

Our results indicate that, as observed for lactose [18], in the case of mannitol the surface physicochemical properties stemming from the different crystal structure represent a tool for modulating carrier-drug interaction and, in turn, aerosolization performance. However, we are aware that a limit of the present approach is the fact that we did not take into account the API polymorphism related to the surface modification often associated with mechanical micronization processes such as jet milling [45]. In fact, in the present work we used a single crystal phase for each of the investigated model drugs. As stated elsewhere [5], the aerosolization process of adhesive mixtures is the result of the summation of several factors which are often interconnected. Our effort consisted in dealing analytically with one of these factors, e.g., the carried solid-state properties. Indeed, the polymorphism of the API represents another fundamental factor that can influence surface energy, aerosolization, flow crystal shape and mechanical properties of the adhesive mixture [46], and will deserve attention for further research works.

## Figures and Tables

**Table 1:** Particle size distribution expressed as equivalent volume diameters (Dv, µm) and span ± standard deviation.

Mannitol	Dv10	Dv50	Dv90	Span
β form	2.90 ± 0.29	11.11 ± 1.75	28.87 ± 1.15	2.34 ± 0.17
δ form	6.21 ± 0.86	23.05 ± 1.85	57.93 ± 1.50	2.24 ± 0.18
α form	6.83 ± 0.42	22.68 ± 2.11	45.36 ± 3.32	2.98 ± 0.75
Hydrate form	6.83 ± 1.17	22.68 ± 2.72	58.14 ± 1.35	1.60 ± 0.07

**Table 2:** Adhesive mixtures of salbutamol sulphate (SS) or budesonide (BUD) with different mannitol carriers and lactose MM50 as the reference. Actual drug content and loaded powder in capsules (mean value ± standard deviation, CV% in parenthesis (*n* = 5).

Carrier	Drug	Drug Content (%)	Loaded Powder Dose (mg)
MM50	SS	0.82 ± 0.03 (3.67)	20.1 ± 0.2 (0.96)
MM50	BUD	0.70 ± 0.02 (2.86)	20.3 ± 0.1 (0.41)
δ form	SS	1.01 ± 0.04 (3.92)	20.1 ± 0.1 (0.26)
δ form	BUD	0.85 ± 0.03 (3.81)	20.0 ± 0.1 (0.38)
β form	SS	0.93 ± 0.03 (3.14)	20.2 ± 0.2 (0.84)
β form	BUD	0.99 ± 0.04 (4.04)	20.0 ± 0.1 (0.32)
α form	SS	0.83 ± 0.04 (4.83)	20.1 ± 0.1 (0.24)
α form	BUD	0.87 ± 0.01 (1.57)	20.0 ± 0.1 (0.26)

**Table 3:** Deposition profiles of salbutamol sulphate from lactose MM50, or mannitol α, β and δ in an ACI after aerosolization with RS01^®^ at 60 L min^−1^ (mean (SD), *n* = 3).

Carrier	Emitted Dose (%)	FPD (µg)	FPF (%)	MMAD (µm)
MM50	80.1 ± 12.7	10.0 ± 2.5	8.4 ± 1.7	3.6 ± 0.1
δ form	90.4 ± 18.5	16.0 ± 1.6	9.3 ± 2.4	4.1 ± 0.0
β form	97.6 ± 3.2	29.8 ± 1.9	13.9 ± 0.7	4.7 ± 0.5
α form	81.7 ± 9.0	21.8 ± 4.8	13.4 ± 4.3	4.1 ± 0.1

**Table 4:** Deposition profiles of salbutamol sulphate from MM50, α, β and δ in an ACI after aerosolization with NESAT^®^ at 48 L min^−1^ [Mean (SD), *n* = 3].

Carrier	Emitted Dose (%)	FPD (µg)	FPF (%)	MMAD (µm)
MM50	96.0 ± 6.2	14.6 ± 1.9	13.5 ± 3.3	3.9 ± 1.2
δ form	95.1 ± 5.6	13.2 ± 1.5	6.5 ± 0.1	5.7 ± 0.4
β form	90.3 ± 12.5	21.0 ± 3.0	11.1 ± 3.7	4.5 ± 0.0
α form	90.5 ± 13.4	18.0 ± 5.5	11.9 ± 6.0	4.0 ± 0.1

**Table 5:** Deposition profiles of budesonide from MM50, α, β and δ in an ACI after aerosolization with RS01 at 60 L min^−1^ [Mean (SD), *n* = 3].

Carrier	Emitted Dose (%)	FPD (µg)	FPF (%)	MMAD (µm)
MM50	87.3 ± 2.0	36.2 ± 0.2	26.6 ± 1.3	1.6 ± 0.2
δ form	78.4 ± 3.5	26.3 ± 8.2	19.6 ± 3.2	2.9 ± 0.4
β form	71.3 ± 7.6	104.7 ± 7.2	58.1 ± 3.9	1.8 ± 0.1
α form	76.5 ± 8.6	84.0 ± 14.6	53.5 ± 5.1	1.8 ± 0.1

**Table 6:** Deposition profiles of budesonide from MM50, α, β and δ in an ACI after aerosolization with NESAT at 48 L min^−1^ [Mean (SD), *n* = 3].

Carrier	Emitted Dose (%)	FPD (µg)	FPF (%)	MMAD (µm)
MM50	76.8 ± 9.7	48.0 ± 2.8	39.1 ± 3.4	2.1 ± 0.2
δ form	71.7 ± 5.0	47.1 ± 10.6	32.0 ± 9.2	1.7 ± 0.1
β form	85.0 ± 8.7	108.0 ± 18.0	57.7 ± 11.8	1.8 ± 0.1
α form	89.5 ± 1.7	87.3 ± 0.8	55.8 ± 1.5	2.0 ± 0.2

## Data Availability

Not applicable.

## Supplementary Materials

The following are available online at https://www.mdpi.com/article/10.3390/pharmaceutics13081113/s1. Figure S1: DSC traces mannitol powders crystallized with different amount of PVP. Figure S2: Dynamic vapor sorption profiles of mannitol powders crystallized with different amount of PVP. Figure S3: DSC traces mannitol powders crystallized with different amount of PVP tested after DVS analysis. Figure S4: FPF of SS and BUD vs. resistance of the device used for aerosolizing adhesive binary mixture with lactose MM50.
