# Supplementary material for: Mannitol Polymorphs as Carrier in DPIs Formulations: Isolation Characterization and Performance
Source: Pharmaceutics. 2021 Jul 21;13(8):1113. doi: 10.3390/pharmaceutics13081113 (PMC8401007; doi:10.3390/pharmaceutics13081113)
Supplement: Supplementary file 1 [file pharmaceutics-13-01113-s001.zip › pharmaceutics-1277302-supplementary.pdf]

pharmaceutics

MDPI

Supplementary Materials: Mannitol Polymorphs as Carrier in DPIs Formulations: Isolation Characterization and Performance

Ayça Altay Benetti, Annalisa Bianchera, Francesca Buttini, Laura Bertocchi and Ruggero Bettini

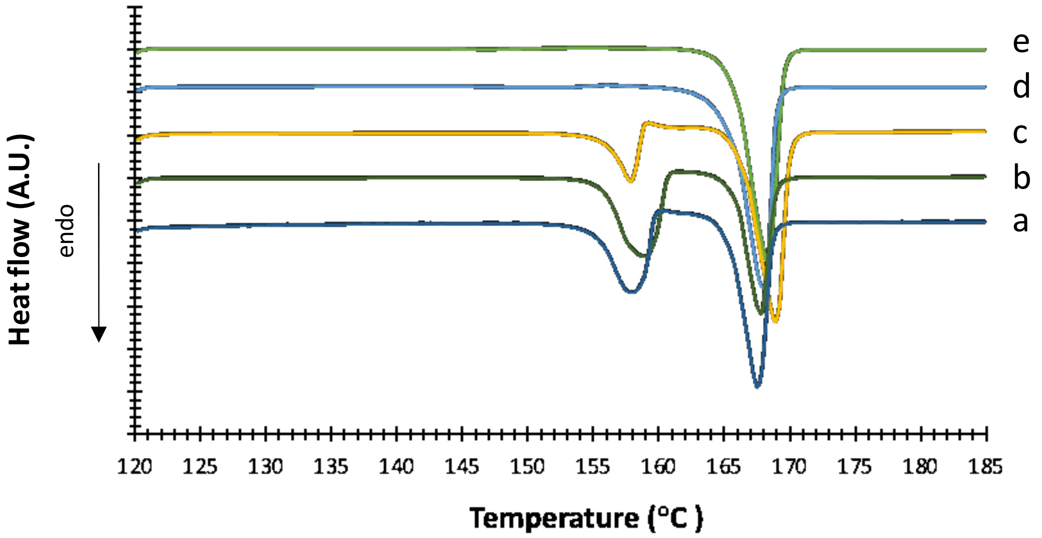

Figure S1. DSC traces mannitol powders supposed to be δ form: crystallized with (a) 3% PVP, (b) 2 % PVP, (c) 1% PVP, (d) 0.5% PVP, (e) without PVP.

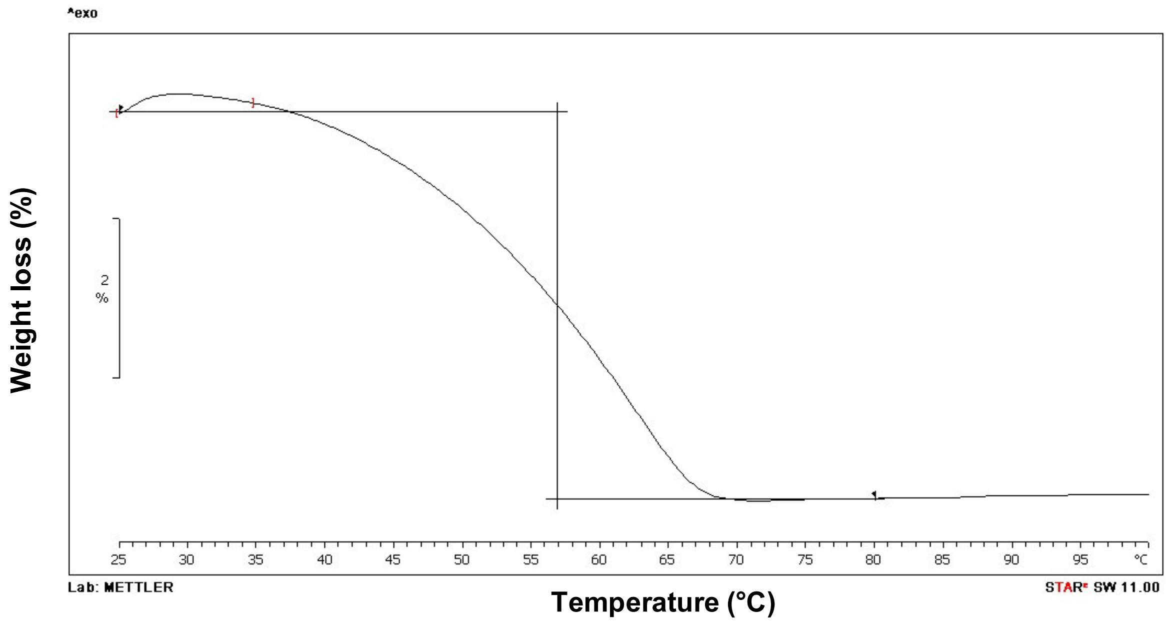

Figure S2. TGA trace of mannitol hemihydrate.

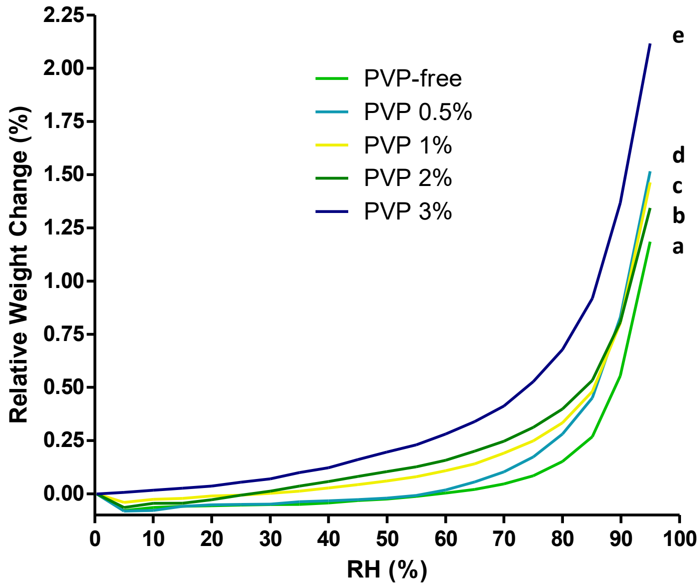

Figure S3. Dynamic vapor sorption curves of mannitol powders supposed to be δ form: crystallized with (e) 3% PVP, (d) 2% PVP, (c) 1% PVP, (b) 0.5% PVP and (a) without PVP K30.

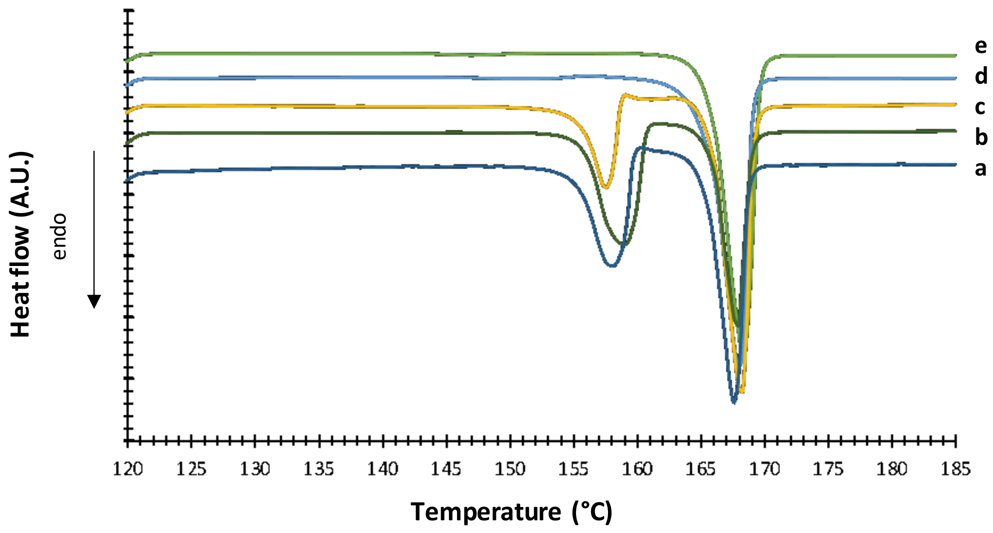

Figure S4. DSC traces mannitol powders supposed to be δ form tested after DVS analysis: crystallized with (a) 3% PVP, (b) 2 % PVP, (c) 1% PVP, (d) 0.5% PVP, (e) without PVP.

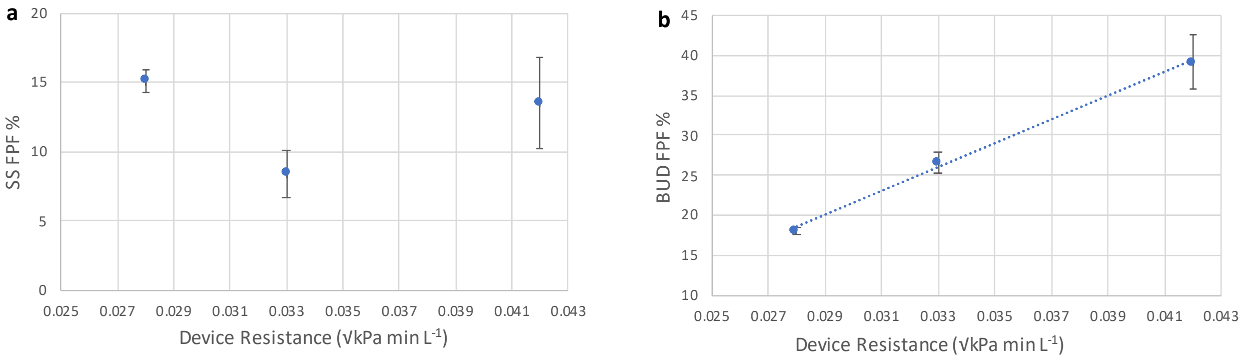

Figure S5. Fine particle fraction of salbutamol sulphate (**a**) and budesonide (**b**) as a function of the resistance of the device used for aerosolizing adhesive binary mixture with lactose MM50. Data for resistance 0.042 and 0.033: present work; data for resistance 0.028: from Della Bella et al. [18].

Table S1. Relevant peaks in supposed α forms with respect to reference from CCDC.

**Alpha CCDC**		**Methanol**		**1% PVA**		**2% PVA**	
**2θ (°)**	**Intensity (cps)**	**2θ (°)**	**Intensity (cps)**	2θ (°)	Intensity (cps)	2θ (°)	Intensity (cps)
8.91	114.7					9.55	810
10.45	75.5					10.8	442.5
13.17	373			12.9	485.5	13.5	253.1
16.77	1000			16.5	495.2	17.1	659.6
18.23	764.9	17.75	264.7	17.95	257	18.55	188.4
19.35	612	19.43	56.5	19.08	931.6	19.7	1000
19.91	691.8	20.08	155.5	19.63	1000	20.25	973.2
20.17	56.4						
20.85	614.8	22.38	1000	20.55	547.1	21.15	462.9
						21.9	1278.5
23.95	13.8	23.65	20.5	22.6	119.7	24.55	1742.5
24.63	328.7	24.9	60	24.33	214.6	24.95	142.6
25.19	53.4						
25.79	33.6			26.18	317.1		
26.51	278.9			26.7	288.3	26.8	27.1
27.03	170.4	27.27	204.6				
27.65	419.9			27.33	343.7	27.35	197.1
28.15	80.9	28.45	297			27.95	192
30.09	65.8			29.58	196.4	30.2	114.6
30.57	43.8	30.57	250	30.7	223.7	31.3	121.4
31.11	64.4	31.65	55.6	32.33	410.7	32.95	217.4
32.65	294.7	32.5	319.4	33.13	257.8	33.75	141.5
33.45	232.7	33.18	25.1	34.75	245.4		

Table S2. Relevant peaks in supposed β forms with respect to reference from CCDC.

**Beta CCDC**		**Beta freeze-drying**		**Beta acetone**	
**2θ (°)**	**Intensity (cps)**	**2θ (°)**	**Intensity (cps)**	**2θ (°)**	**Intensity (cps)**
10.11	116.1			9.64	434.3
11.09	27.8			10.46	104.8
14.27	461.2	13.14	190.1	14.64	344.7
16.41	354.3	16.73	718.2	17.89	98.1
18.41	878.7	18.21	539.9	18.74	780.7
18.71	157.1				
19.29	36.8	19.31	749.6	19.46	109.2
20.09	422.1	19.86	1000	20.41	436.5
20.77	589.3	20.71	738.4	21.09	425.2
21.33	282.8			21.66	75.3
22.09	9.1				

23.07	1000	22.84	442	23.39	1000
24.35	154.2	24.61	272.4	24.64	181.9
25.55	140.8	26.44	233.9	25.22	133.9
26.21	76.7	26.96	225.7	25.89	110.8
27.99	111.7	27.59	323.7	28.29	150.4
29.15	306.1	28.94	140.5	29.49	371
30.21	25.5				
31.01	58.4	30.94	162		
31.43	61.8			31.77	122
32.35	80.9			32.66	104
33.25	172.3	32.59	298.9		
33.47	123.8	33.39	212.5	33.54	269
33.89	238.4				
34.59	63.8			34.62	66

Table S3. Relevant peaks in supposed δ forms with respect to reference from CCDC.

Delta CCDC		Acetone		0.5% PVP		1% PVP		2% PVP		3% PVP	
2θ (°)	Intensity (cps)	2θ (°)	Intensity (cps)	2θ (°)	Intensity (cps)	2θ (°)	Intensity (cps)	2θ (°)	Intensity (cps)	2θ (°)	Intensity (cps)
9.35	773.3	9.38	1000	9.5	1000	9.33	1000	9.25	1000	9.18	419.2
		16.93	36.2	13.43	43.6						
				17.05	142.9						
				18.5	45.4						
19.11	110.6	19.1	314.9	19.25	252	19.05	341.1	18.98	483	18.9	308.7
19.47	82					19.98	847.3	19.93	874.2	19.83	1000
20.07	1000	20.05	905	20.18	860.5						
20.75	343.8	20.75	232.9	20.9	321.9	20.7	262.4	20.65	265.1	20.58	357.7
20.91	306.6										
21.75	330	21.7	402.2	21.85	345.3	21.65	390.9	21.58	426.5	21.5	472.4
22.39	44.2	22.35	24.9	22.48	30.2	22.3	37.4	22.25	38.9	22.18	47.3
22.55	22.8	22.98	20.8								
24.33	411.1	24.3	548.7	24.45	476.3	24.25	532.4	24.18	638.6	24.1	667.5
25.05	378.4	24.9	425.8	25.02	477.1	24.85	520.8	24.77	583.8	24.73	749.2
27.55	138.4	27.5	204	27.65	197.8	27.45	200.1	27.38	252.8	27.3	269.6
28.19	11.8			28.25	29.3	28	15.4	27.98	23.3	27.88	25.5
29.01	23.5	29	57.5	29.15	36.2	28.95	57.2	28.85	88.8	28.77	73.1
31.83	44.6	31.73	68.9	31.85	67.1	31.68	80.3	31.6	106.6	31.52	114.6
34.63	100.7	34.65	85.5	34.75	67.7	34.55	120.1			34.43	197.4

Table S4. Relevant peaks in supposed hydrate forms with respect to reference from CCDC.

Idrato CCD		Without $CaCl_2$		With $CaCl_2$	
2θ (°)	Intensity (cps)	2θ (°)	Intensity (cps)	2θ (°)	Intensity (cps)
9.54	1000	9.19	1000	9.25	1000
9.94	68.4				
10.36	83.5				
		14.75	814	14.85	970
16.56	164.2	16.16	235.5	16.18	182.9
17.3	58.1	17.54	678.4	17.55	472.9
17.98	911.1	18.44	518.8	17.58	472.4
19	637.4	18.81	210.7	18.58	294.3
19.4	568.3	19.54	583.8	19.58	389.7
19.98	512.5	20.06	288	20.18	201.4
20.8	138	20.74	225.9		
21.76	104.3	22.66	411.5	22.8	298
23.14	514.9	23.01	258.7		
25.24	187.4	24.84	409.6		
25.86	258.2	25.31	538.4	25.38	503.5
26.08	102.5	26.61	425.8	26.68	351.1

27.26	416.7	28.34	286.2	28.28	270.5
27.76	66.6				
28.46	92.3				
28.98	229.7	29.11	155.9		
30.56	68.5				
32.84	191.5	32.31	195.1		
33.78	27.3	33.13	191.9	33.16	154.6
34.1	109.7	34.21	422.8	34.21	482.3
34.66	123.5				

Table S5. Relevant peaks in α forms after 1 month, 1 year and 2 years of storage at 40°C 75% RH with respect to reference from CCDC .

**Alpha CCDC**		**At preparation**		**After 1 month**		**After 1 year**		**After 2 years**	
**2θ (°)**	**Intensity (cps)**	**2θ (°)**	**Intensity (cps)**	**2θ (°)**	**Intensity (cps)**	**2θ (°)**	**Intensity (cps)**	**2θ (°)**	**Intensity (cps)**
8.91	114.7					9.18	113.2		
10.45	75.5								
13.17	373	13.5	253.1	13.98	183.6	13.38	230.8	13.9	292.1
				14.65	154				
16.77	1000	17.1	659.6	17.52	1000	16.95	1000	17.5	1000
18.23	764.9	18.55	188.4			18.6	169.2	18.95	655.4
19.35	612	19.7	1000			19.8	441.5		
19.91	691.8	20.25	973.2	19	300.9				
20.17	56.4			20.18	468.3			20.08	628
20.85	614.8	21.15	462.9	20.7	425.3	21	749.2	20.63	717.4
				21.55	385.1			21.5	897.6
23.95	13.8							23.6	201.7
24.63	328.7	24.95	142.6	25.43	181.6				
25.19	53.4					25.33	76.1	25.33	299.5
25.79	33.6			26.08	79.6				
26.51	278.9	26.8	27.1						
27.03	170.4			27.3	164.5	27.2	192.2	27.2	281.7
27.65	419.9	27.35	197.1			27.98	104.9	27.73	231.3
28.15	80.9	27.95	192	27.8	110.2			28.33	326.2
30.09	65.8	30.2	114.6			30.83	76.8		
30.57	43.8	31.3	121.4	28.4	134.2				
31.11	64.4	32.95	217.4					31.73	130
32.65	294.7	33.75	141.5	33.43	208.9				
33.45	232.7			34.23	139.2			33.33	381.5
35.07	117.5							34.13	348.7

Table S6. Relevant peaks in δ forms after 1 month, 1 year and 2 years of storage at 40°C 75% RH with respect to reference from CCDC .

**Delta CCDC**		**At preparation**		**After 1 month**		**After 1 year**		**After 2 years**	
**2θ (°)**	**Intensity (cps)**	**2θ (°)**	**Intensity (cps)**	**2θ (°)**	**Intensity (cps)**	**2θ (°)**	**Intensity (cps)**	**2θ (°)**	**Intensity (cps)**
9.35	773.3	9.33	1000			9.7	1000	10.08	482.6
								10.88	30.5
				13.95	66.5			15.08	49
				18.05	130.5			19.2	158.1
19.11	110.6	19.05	341.1	18.75	160.6			19.9	163.8
19.47	82	19.98	847.3	19.7	1000	19.47	262.7	20.83	1000
20.07	1000								
20.35	89.1								
20.75	343.8	20.7	262.4	20.43	256.1	20.37	906.9	21.52	303.3
20.91	306.6								
21.75	330	21.65	390.9	21.38	451.2	21.04	403.2	22.5	412.6
22.39	44.2	22.3	37.4	22.65	211.2	22.02	379.1	23.2	33.8

22.55	22.8					22.62	48	23.85	183.7
24.33	411.1	24.25	532.4	23.95	602.2	24.64	578.7	25.08	516.7
25.05	378.4	24.85	520.8	24.58	320.6	25.2	529.7	25.68	388.2
27.55	138.4	27.45	200.1	27.15	249.3	27.85	178.2	28.3	183.1
28.19	11.8	28	15.4	27.52	58.1	28.5	22.5	29.9	83.2
29.01	23.5	28.95	57.2	28.73	129.2	29.37	38.8	32.5	59.9
31.83	44.6	31.68	80.3	31.4	60.3	32.02	78.8	34.02	69.6
				32.85	77.4				
34.63	100.7	34.55	120.1	34.25	170.6				
34.95	92.4	34.88	52.3						
